# Contextual Regulation of the Kynurenine Pathway and Its Relevance for Personalized Psychiatry

**DOI:** 10.3390/jpm16020118

**Published:** 2026-02-14

**Authors:** Stephen Murata, Gregory Oxenkrug, Angelos Halaris

**Affiliations:** 1 Pine Rest Christian Mental Health Services, Michigan State University, 300 68th Street SE, Grand Rapids, MI 49548, USA; 2 Department of Psychiatry, Tufts University School of Medicine, Boston, MA 02111, USA; 3 Department of Psychiatry and Behavioral Neurosciences, Loyola University Chicago, Maywood, IL 60153, USA

**Keywords:** kynurenine pathway, tryptophan metabolism, neuroinflammation, kynurenine/tryptophan ratio, quinolinic acid, immune–metabolic interactions, neuroprogression, personalized psychiatry

## Abstract

The kynurenine pathway (KP) is the primary route of tryptophan metabolism and a key interface linking immune activation, metabolic state, and neurochemical signaling. Although KP biomarkers are widely studied in psychiatric disorders, their interpretation remains inconsistent, in part due to biological context and compartmentalization. In this narrative review, we integrate evidence across peripheral and central systems to clarify how age, sex hormones, metabolic health, inflammation, and behavioral factors systematically bias KP flux and shape biomarker readouts. We re-examine the interpretation of the kynurenine/tryptophan ratio in light of differential IDO1 and TDO2 regulation, blood–brain barrier constraints, and cell-specific downstream metabolism that governs neuroprotective and neurotoxic outputs. We further synthesize clinical evidence linking KP alterations to symptom severity, cognitive dysfunction, treatment resistance, and suicidality, highlighting quinolinic acid as a mechanistic node connecting immune activation to glutamatergic dysregulation. Together, this framework reframes the kynurenine pathway not as a static biomarker of disease, but as a context-sensitive metabolic system with direct implications for study design, risk stratification, and personalized approaches in psychiatry.

## 1. Introduction: Towards a Personalized Psychiatry

Psychiatry is undergoing a shift from categorical diagnostics toward mechanistically grounded, biologically stratified approaches [1,2]. This shift reflects growing recognition that mental disorders are heterogeneous and arise from complex interactions among multiple systems. Dimensional models expand clinical reasoning beyond symptom clusters to incorporate biological domains such as inflammation and immunology, hypothalamic–pituitary–adrenal (HPA) axis function, serotonergic signaling, and glutamatergic neurotransmission [3,4,5]. Meanwhile, there is a need to account for the temporality—the chronic, relapsing–remitting course of illness which gives rise to the concept of neuroprogression [6,7]. Balancing these perspectives is essential for translating biology into personalized care.

**Figure 1:**
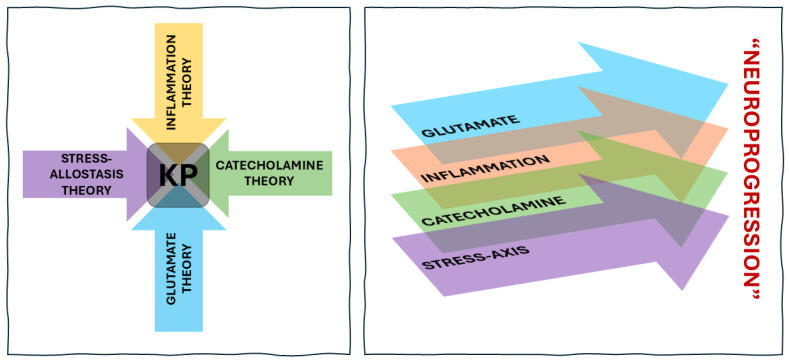
Traditional theories of depression—including stress–allostasis, inflammation, catecholamine dysregulation, and glutamatergic dysfunction—are often treated as independent explanatory models (left panel). The kynurenine pathway (KP) sits at the intersection of these domains, integrating immune activation, hypothalamic–pituitary–adrenal (HPA) axis signaling, monoamine metabolism, and excitatory–inhibitory balance through its regulation of tryptophan degradation and production of neuroactive metabolites. The right panel illustrates how these interacting systems overlap and accumulate across time, contributing to neuroprogression—the progressive consolidation of biological alterations underlying chronic or recurrent illness.

The kynurenine pathway (KP) represents an integrative biological axis linking immune, metabolic, and neurotransmitter systems in the regulation of brain function [8]. As the primary route of tryptophan degradation, it yields neuroactive metabolites whose balance shifts with inflammatory, hormonal, and cellular signals, redirecting flux between neuroprotective (KYNA) and neurotoxic (QUIN) branches and modulating glutamatergic tone. Differential enzyme expression across peripheral tissues and CNS cell types further embeds the pathway within interactions among systemic inflammation, BBB transport, and local microglial and astrocytic metabolism. Clinically, meta-analyses demonstrate elevated KYN/TRP ratios in major depressive and bipolar disorders [9,10], altered 3-HK/KYNA reflecting neurotoxic–neuroprotective balance, and pronounced QUIN–KYNA abnormalities in suicidality [11], with pregnancy–postpartum studies highlighting state-dependent shifts [12]. Together, these findings position the kynurenine pathway as a mechanistic bridge linking peripheral immune signals to central neurotransmission and behavioral change, within a broader network of interacting immune, metabolic, monoaminergic, and glutamatergic processes (Figure 1).

In this review, we describe the biological architecture of the kynurenine pathway (KP) and its relevance to personalized psychiatry. Rather than serving as a static biomarker of depression or inflammation, the KP functions as a dynamic, compartmentalized system that regulates the balance between neurotoxic and neuroprotective signaling. We outline key principles of tryptophan metabolism—including peripheral–central compartmentalization, blood–brain barrier transport, and cell-specific enzyme expression—that govern KP flux toward protective or toxic outputs. We then examine how immune, metabolic, and stress-related inputs bias KP enzyme activity, particularly toward quinolinic acid-dominant profiles, reflecting reduced buffering against neurotoxicity that may accumulate across illness stages and contribute to neuroprogression. Finally, we integrate evidence linking KP alterations to transdiagnostic clinical dimensions—including inflammatory endophenotypes, suicidality, cognitive dysfunction, and treatment resistance—to show how KP profiles define biologically meaningful subtypes with implications for disease course and treatment stratification.

## 2. Biology of the Kynurenine Pathway

### 2.1. TRP Handling and Compartmentalization

**Figure 2:**
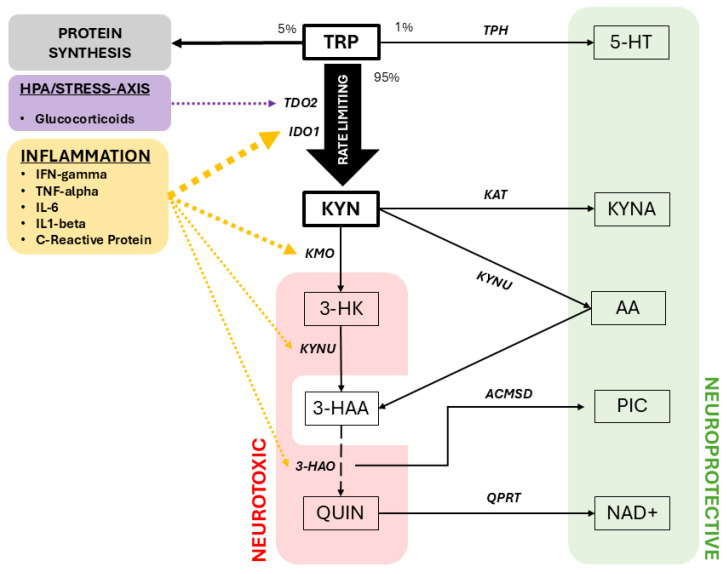
**Overview of tryptophan metabolism through the kynurenine pathway.** Tryptophan (TRP) is primarily metabolized via the kynurenine pathway (KP), with indoleamine 2,3-dioxygenase 1 (IDO1) and tryptophan 2,3-dioxygenase (TDO2) serving as rate-limiting enzymes. Inflammatory mediators (e.g., IFN-γ, TNF-α, IL-6) and stress-related signals influence upstream KP activation, diverting TRP away from serotonin synthesis and protein production. Downstream metabolism yields neuroactive metabolites with divergent functional properties, including kynurenic acid (KYNA) and quinolinic acid (QUIN), as well as intermediates contributing to NAD^+^ biosynthesis. **KP metabolites:** AA, anthranilic acid; 3-HAA, 3-hydroxyanthranilic acid; 3-HK, 3-hydroxykynurenine; KYNA, kynurenic acid; KYN, kynurenine; NAD^+^, nicotinamide adenine dinucleotide; PIC, picolinic acid; QUIN, quinolinic acid; TRP, tryptophan; 5-HT, serotonin. **KP enzymes:** ACMSD, aminocarboxymuconate semialdehyde decarboxylase; IDO1, indoleamine 2,3-dioxygenase 1; KAT, kynurenine aminotransferase; KMO, kynurenine 3-monooxygenase; KYNU, kynureninase; QPRT, quinolinate phosphoribosyltransferase; TDO2, tryptophan 2,3-dioxygenase; TPH, tryptophan hydroxylase.

Tryptophan (TRP) is an essential amino acid whose metabolic fate is partitioned among protein synthesis, serotonin (5-HT) production, and the kynurenine pathway (KP)—with ~95% shunted through the KP, ~5% incorporated into protein, and only ~1% available for CNS serotonin synthesis [13,14]. In circulation, ~90% of TRP is albumin-bound [15]; although inflammation lowers albumin and transiently increases free TRP [16], this does not translate into greater CNS availability, because TRP must cross the blood–brain barrier (BBB) via the LAT1 transporter, where it competes with other large neutral amino acids [17]. This competitive gating represents the first major regulatory distinction between peripheral and central TRP handling. An overview of tryptophan metabolism through the kynurenine pathway, including its major fates and rate-limiting steps, is shown in Figure 2.

5-HT synthesis is also regulated differently across compartments [18]. TPH1, expressed in enterochromaffin cells and platelets, drives peripheral 5-HT production, whereas TPH2 in the raphe nuclei governs CNS 5-HT synthesis and depends on the cofactor BH4 [19]. Peripheral 5-HT cannot cross the BBB, and central synthesis is constrained both by TRP transport and by TPH2 regulation. These nuances challenge earlier “5-HT deficit” models of depression: while monoaminergic dysfunction is clinically meaningful, evidence increasingly supports a model of serotonergic dysregulation—altered synthesis, release, reuptake, and receptor sensitivity—rather than a uniform substrate deficiency.

Adding further complexity, most antidepressants reach the brain through passive diffusion with variable BBB penetration, while the 5-HT transporter (SERT)—the primary target of SSRIs—is expressed widely in raphe neurons, but also on cortical, limbic, and striatal terminals, and even peripherally in platelets and some immune cells. Genetic variation in the SERT promoter (5-HTTLPR L vs. S alleles) influences expression levels and may modulate treatment response or stress sensitivity, though its effects are modest and context-dependent [20]. Together, these layers of regulation—TRP transport, compartment-specific 5-HT synthesis, SERT distribution, and dynamic modulation across states—underscore why contemporary models emphasize serotonergic dysregulation within broader network and immune–metabolic contexts rather than a simple deficit framework.

### 2.2. Rate-Limiting Control: TDO2 vs. IDO1

The initial, rate-limiting conversion of TRP to kynurenine (KYN) is mediated by two enzymes—tryptophan 2,3-dioxygenase (TDO2) and indoleamine 2.3-dioxygenase (IDO1). TDO2 and IDO1 differ in their tissue distribution and regulatory inputs. In peripheral tissues, KP activation is driven largely by TDO2 expressed in hepatocytes, and induced by glucocorticoids, stress hormones, and TRP availability [21,22]. TDO2 activation may help regulate systemic TRP homeostasis and, in doing so, can influence systemic TRP availability for central 5-HT synthesis, although it produces relatively modest amounts of downstream neuroactive KP metabolites.

In CNS, KP activation is regulated by IDO1 expressed in microglia, infiltrating macrophages, dendritic cells, and certain endothelial and astroglial populations [23]. IDO1 expression is typically upregulated by pro-inflammatory cytokines—particularly IFN-γ, with contributions from TNF-α, IL-1β, and type I interferons—as well as by innate immune signals such as TLR agonists [24,25]. Because this induction occurs within immune and glial populations, IDO1-driven KP metabolism may give rise to a different downstream profile, including increased production of metabolites such as 3-hydroxykynurenine (3-HK) and quinolinic acid (QUIN) which implicate redox balance and glutamatergic signaling [26]. In a sense, TDO2- and IDO1-mediated KP activation appear to reflect partially distinct physiological states—stress-related metabolic regulation versus cytokine-associated immune activation—which may have different implications for TRP availability, 5-HT synthesis, and neurobiological outcomes.

### 2.3. Peripheral KYN/TRP as a Context-Dependent Marker of KP Activation

We now turn to the interpretation of the peripheral KYN/TRP ratio, which—by indexing sequential metabolites at the first rate-limiting step—has been widely used as an indirect marker of kynurenine pathway activation. As alluded to above, the differential regulation of KP activation by IDO1 and TDO2 invites nuanced biological interpretation of the peripheral KYN/TRP ratio. It is plausible that, under basal conditions, KYN/TRP may primarily reflect hepatic TDO2-regulated tryptophan homeostasis [27], whereas with immune activation KYN/TRP shifts toward an IDO1-driven marker of cytokine-induced tryptophan catabolism [25].

This distinction is relevant in mood disorders, where systemic inflammatory markers are variably elevated in major depressive disorder and bipolar disorder, and where efforts to define a high-inflammation endophenotype (e.g., hsCRP > 3 mg/L) have linked inflammation to greater symptom burden, cognitive impairment, and reduced antidepressant responsiveness [28,29,30,31]. Consistent with this, meta-analyses report elevated KYN/TRP ratios at the group level in both disorders [9,10]; however, associations with depressive symptoms and diagnostic status remain heterogeneous, and KYN/TRP abnormalities may persist independent of illness phase [32,33].

### 2.4. Blood–Brain Barrier Constraints and Central Compartmentalization of the KP

**Figure 3:**
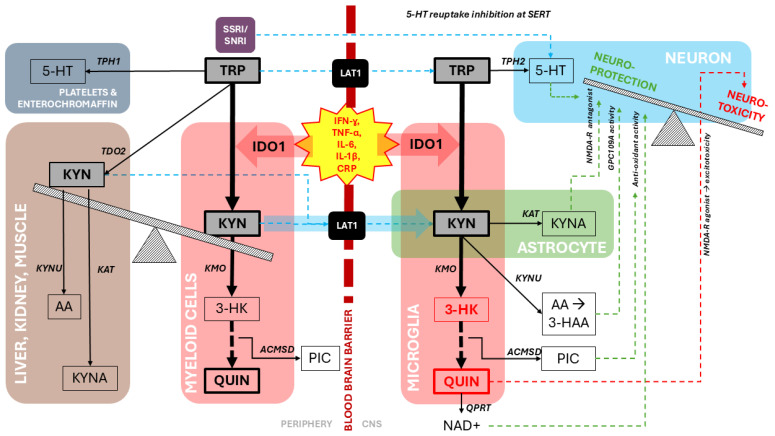
Compartmentalized regulation of tryptophan metabolism and kynurenine pathway flux linking systemic inflammation to central neurotoxicity. Pro-inflammatory cytokines robustly induce IDO1 and KMO in peripheral immune and tissue cells, redirecting TRP toward KYN and the neurotoxic KP branch. KYN—unlike downstream metabolites—crosses the blood–brain barrier via LAT1 and supplies substrate for CNS KP metabolism. Within the brain, astrocytes preferentially generate neuroprotective KYNA, whereas microglia—expressing high IDO1/KMO and low QPRT—produce 3-HK and QUIN, shifting the KYNA–QUIN balance toward excitotoxicity. QUIN may contribute to neuronal injury directly (NMDA-R agonism, oxidative stress) or enter the de novo NAD^+^ pathway via QPRT. Serotonin synthesis pathways (TPH1/TPH2) and the pharmacologic action of SSRIs/SNRIs are shown as parallel TRP-utilization routes. Together, the diagram illustrates how systemic inflammatory signals reshape TRP handling, enhance central KYN influx, and bias CNS metabolism toward neurotoxic KP outputs.

In the context of IDO1-mediated KP activation in the periphery, interpretation of central effects must account for blood–brain barrier (BBB) constraints. Under physiological conditions, only tryptophan (TRP) and kynurenine (KYN) efficiently cross the BBB via the large neutral amino acid transporter LAT1 [34]. Except in cases of marked BBB disruption—where passive diffusion of downstream metabolites may occur [35]—most KP metabolites are effectively impermeant and therefore reflect local glial metabolism within the CNS. Given that approximately 40% of brain KYN is peripherally derived, a proportion that increases substantially during inflammation [36], elevated peripheral KYN/TRP may plausibly index concentration-dependent KYN influx into the brain, where subsequent metabolism is locally regulated. The compartment-specific organization of kynurenine pathway metabolism across the periphery, blood–brain barrier, and central nervous system is summarized in Figure 3.

This compartmentalization imposes important limits on the interpretation of peripheral KP measures. Concordance between peripheral and central compartments is metabolite-specific and incomplete: CSF studies demonstrate larger effect sizes for quinolinic acid (QUIN) and kynurenic acid (KYNA) than blood-based measures across mood and psychotic disorders [37], while brain–blood correspondence is strongest for KYN and 3-hydroxykynurenine and weak or inconsistent for TRP, KYNA, and QUIN [34]. Accordingly, peripheral KP markers should not be viewed as direct surrogates of CNS biochemistry. Nonetheless, peripheral KP alterations show independent associations with psychiatric symptoms and white matter abnormalities consistent with neuroprogressive trajectories [33,34,38,39,40]. Thus, although peripheral KP signals only partially reflect central metabolism, they may capture broader systemic and neurobiological processes relevant to structural brain change and illness progression [41]. For clarity, key aspects of KP compartmentalization, buffering capacity, and measurement considerations are summarized in Table 1.

### 2.5. Neuroactive Metabolites Downstream of KP Activation

Although serotonin (5-HT) has historically occupied a central place in models of depression, only a small fraction of tryptophan (TRP) is available for central 5-HT synthesis, with the majority metabolized through the kynurenine pathway (KP). Early formulations of serotonin deficiency, articulated by Coppen and later elaborated by Åsberg and van Praag, focused primarily on serotonergic tone itself [48]. Building on this framework, Lapin and Oxenkrug proposed in 1969 that a metabolic shunt of TRP from the serotonin pathway toward the KP could contribute to serotonergic deficiency in depression. At the time, kynurenines were considered largely inert intermediates of the TRP–NAD^+^ pathway; Lapin, however, hypothesized that they possessed intrinsic neuroactive properties [49]. This hypothesis was subsequently supported by experimental evidence showing that kynurenine and several downstream metabolites—including 3-hydroxykynurenine, anthranilic acid, 3-hydroxyanthranilic acid, picolinic acid, quinolinic acid, and nicotinic acid—attenuate central serotonergic processes in vivo [47,50], establishing the concept of neuroactive kynurenines and later summarized in historical reviews by Oxenkrug and colleagues [51,52].

Conceptualizing KP activation as a 5-HT shunt therefore remains a biologically and historically grounded entry point: conditions that increase KP flux—such as immune activation, stress, or metabolic signaling—constrain serotonergic synthesis while simultaneously increasing the availability of kynurenine for further metabolism. Importantly, this diversion represents a metabolic commitment rather than a terminal mechanism. Once TRP is routed into the KP, its neurobiological impact is determined not only by reduced serotonergic output but by how kynurenine is subsequently metabolized across peripheral and central compartments.

Downstream KP metabolism diverges into multiple, cell-specific branches that generate metabolites with distinct—and sometimes opposing—effects on NMDA receptor signaling, oxidative stress, microglial activation, and synaptic plasticity. These branches are often described as “neuroprotective” and “neurotoxic,” exemplified by kynurenic acid (KYNA) and quinolinic acid (QUIN) [53,54]. However, this dichotomy is inherently context dependent, varying with regional enzyme expression, redox state, inflammatory cues, and illness stage. Accordingly, the KP is best understood as a distributed regulatory network, in which shifts in downstream flux shape vulnerability to neuroinflammation, excitotoxicity, and mood dysregulation.

### 2.6. The Initial Branch—The Trifurcation of KYN

Kynurenine (KYN) can be converted to kynurenic acid (KYNA) by kynurenine aminotransferases (KATs), or to 3-hydroxykynurenine (3-HK) by kynurenine 3-monooxygenase (KMO) [55]. Rather than strict “branch points,” these routes reflect which enzymes are present in a given tissue. In the CNS, astrocytes predominantly express KATs and therefore produce more KYNA, a metabolite with neuroprotective, NMDA-antagonist, and anti-inflammatory effects [56]. Microglia and infiltrating macrophages, by contrast, express KMO and generate 3-HK and quinolinic acid (QUIN), which contribute to oxidative stress and excitotoxicity [57,58]. A similar pattern appears in peripheral immunity: monocytes and macrophages are KMO-rich, while liver, kidney, and other tissues express both KMO and KATs to varying degrees [21]. This compartment-specific enzyme distribution—more than a single fork in the pathway—shapes whether KP activation leads toward protective buffering or toward neuroinflammatory and excitotoxic processes.

### 2.7. Neuroprotective Branches: Kynurenic Acid (KYNA) and Anthranilic Acid (AA)

KYNA is an endogenous modulator of glutamatergic neurotransmission. In the mammalian brain, KYNA acts as a broad-spectrum antagonist at the glycine co-agonist site of the NMDA receptor and modulates other glutamate-related targets, contributing to protection against excitotoxic injury and glutamate-driven neurotoxicity [26]. Consistent with this role, convergent clinical data suggest that relatively low KYNA—or a shift away from KYNA production—tracks with greater depressive symptom burden, cognitive dysfunction, and suicidality in subsets of mood and psychotic disorders [11,59].

KP metabolite AA is able to cross the blood–brain barrier (BBB) by passive diffusion [43]. Thus, peripheral AA (along with KYN, which can also freely pass the BBB) is an important window or proxy into CNS-related KP metabolism [60]. The mechanism of AA and related derivatives is an area of active exploration. There is an emerging line of inquiry into whether AA and its downstream metabolites may mediate immunomodulatory effects, in part through its putative agonism at the G protein-coupled receptor 109A (GPR109A) which regulates inflammation and demyelination [61,62]. Current evidence suggests that AA exerts significant anti-inflammatory effects by downregulating proinflammatory cytokines and suppressing microglial activation, thereby reducing neuroinflammation in the CNS [63,64]. Additionally, AA provides neuroprotection by preventing neuronal damage from excitotoxicity and oxidative stress, while enhancing the expression of neuroprotective factors [65,66]. In a recent multi-site study, high peripheral AA/ICAM1 at baseline predicted ketamine remission in TRD [67], which supports the emerging relevance of AA and the potential utility of combined KP-immune–metabolic composite markers.

### 2.8. Initial Neurotoxic Branch: 3-Hydroxykynurenine (3-HK)

The neurotoxic limb of the pathway is dominated by 3-hydroxykynurenine (3-HK) and quinolinic acid (QUIN). 3-HK, produced from KYN by kynurenine monooxygenase (KMO), is a potent pro-oxidant molecule. Experimental studies show that 3-HK undergoes redox cycling, generates reactive oxygen species, and promotes lipid peroxidation and oxidative damage in neural tissues, particularly under inflammatory conditions [26]. Clinically, elevated 3-HK levels—or a shift toward 3-HK-dominated profiles—have been linked to greater symptom severity and cognitive impairment in mood and psychotic disorders, especially when considered relative to KYNA [54].

KMO is the critical ‘gatekeeping’ enzyme for the neurotoxic branch. By converting KYN to 3-HK, KMO diverts substrate away from KYNA and PIC toward reactive oxygen species generation and QUIN production. Preclinical KMO-inhibition studies and genetic/expression data in neurodegenerative and psychiatric contexts support the idea that increased KMO activity shifts KP balance toward toxicity and exacerbates excitotoxic and inflammatory cascades [26]. KYNU, acting downstream on several intermediates, further shapes neurotoxic versus neuroprotective output. Alterations in KYNU expression and genetic variation have been implicated in subgroups with elevated suicide risk [68], though evidence remains less consistent than for KMO and ACMSD at this time.

### 2.9. Downstream Diversion from Neurotoxicity: Picolinic Acid (PIC)

Picolinic acid (PIC) is generated when ACMSD diverts flux away from quinolinic acid (QUIN), and its metal-chelating and antioxidant properties may buffer against QUIN-driven excitotoxicity [69]. Cerebrospinal fluid studies in suicide attempters show elevated QUIN on a background of low PIC, with the PIC/QUIN ratio proposed as an index of neuroprotective capacity and suicidal vulnerability [70]. The integrity of this neuroprotective arm depends on both KAT and ACMSD activity. Kynurenine aminotransferases (KATs) convert KYN to kynurenic acid (KYNA), and increasing KYNA in preclinical models reduces glutamate-mediated excitotoxicity and modulates dopaminergic and cognitive phenotypes, positioning glial KATs as metabolic gates on excitatory tone (Schwarcz et al., 2012) [26]. ACMSD, in turn, governs whether ACMS is shunted toward PIC or proceeds to QUIN, and reduced ACMSD expression or functional variants have been linked to higher central QUIN burden and suicidal behavior, underscoring its role in determining neurotoxic versus neuroprotective balance [70].

### 2.10. Quinolinic Acid (QUIN) and Concepts of Neurotoxicity and Excitotoxicity

Quinolinic acid (QUIN), produced downstream of 3-hydroxyanthranilic acid, is an endogenous NMDA receptor agonist and one of the best-characterized excitotoxins in the central nervous system. Under inflammatory conditions, QUIN is generated predominantly by activated microglia and infiltrating macrophages, positioning it at the interface of immune activation and glutamatergic signaling [45,46]. In this context, excitotoxicity refers not merely to excessive synaptic excitation, but to sustained NMDA receptor overactivation leading to pathological calcium influx, mitochondrial dysfunction, reactive oxygen and nitrogen species generation, and downstream injury to neurons, oligodendrocytes, and astrocytes [71]. QUIN further amplifies these effects by impairing astrocytic glutamate uptake and promoting glutamate release, thereby increasing extracellular glutamate and prolonging NMDA receptor stimulation [72].

Beyond receptor agonism, QUIN contributes to neurotoxicity through multiple convergent mechanisms, including lipid peroxidation, DNA damage, and disruption of cellular energy metabolism [46]. These effects are particularly relevant in microglia-rich and metabolically vulnerable brain regions, where QUIN accumulation may bias local circuits toward excitatory–inhibitory imbalance and synaptic destabilization. Consistent with this framework, inflammatory states associated with elevated QUIN are accompanied by alterations in glutamate metabolism and increased extra-synaptic glutamate, a pattern observed in both preclinical models and human studies of inflammation-associated depression [41,73].

Clinically, elevations in QUIN have been associated with a range of severe and treatment-refractory psychiatric phenotypes characterized by heightened inflammatory burden, glutamatergic dysregulation, and cognitive or affective instability [3,11,45]. Within this spectrum, QUIN increases are particularly pronounced in the context of suicidality, which has emerged as one of the most robust and reproducible clinical correlates of neurotoxic KP bias. Increased cerebrospinal fluid QUIN has been reported in suicide attempters, with levels correlating with suicide intent severity [59]. Subsequent studies by Brundin and colleagues, as well as integrative analyses by Bryleva and Brundin, confirmed that suicidal patients frequently exhibit a shift in KP metabolism toward QUIN and other neurotoxic metabolites, consistent with heightened excitotoxic and inflammatory load [11,70]. Importantly, these findings do not suggest a suicide-specific mechanism, but rather highlight suicidality as a clinically tractable endpoint of more generalized QUIN-associated processes, including glutamatergic excess, circuit instability, and neural injury. Together, this body of work implicates QUIN not only as a downstream marker of KP activation, but as a mechanistic contributor to neurobiological severity across psychiatric illness.

### 2.11. Composite KP Ratios as Integrated Readouts of KP Balance

Because these metabolites function in counter-balancing ways, simple absolute concentrations often obscure the biologically relevant signal. Over the last decade, multiple groups have emphasized composite ratios as more informative readouts of KP state.

A commonly used composite marker is the 3-HK/KYNA ratio, which reflects the balance between the oxidative “neurotoxic” branch (3-hydroxykynurenine) and the protective “neuroactive” branch (kynurenic acid) of the kynurenine pathway. In bipolar disorder, there seems to be a shift toward increased kynurenine breakdown along the neurotoxic branch, with higher 3-HK/KYNA ratios correlating with specific symptom dimensions and, in some analyses, poorer cognitive performance [54]. Similar patterns—elevated 3-HK/KYNA or related toxic/protective ratios—have been observed in major depressive disorder and other conditions, where they have been linked to greater depressive severity, suicidality, and treatment resistance in smaller cohorts [11].

The KYNA/QUIN ratio is frequently used as an index of neuroprotective versus neurotoxic balance. In MDD, Savitz et al. reported reduced KYNA and elevated QUIN in CSF, resulting in a lower KYNA/QUIN ratio in both acutely depressed and remitted patients compared with controls, consistent with a persistent shift toward QUIN dominance [32]. In suicidality, Erhardt et al. found that suicide attempters had elevated CSF QUIN and a significantly increased QUIN/KYNA quotient, linking a more ‘toxic’ ratio profile to suicidal behavior and inflammatory markers such as CSF IL-6 [59]. Bryleva and Brundin’s review synthesizes these findings and highlights reduced KYNA/QUIN as a key signature of suicide-related KP dysregulation [11].

The PIC/QUIN ratio has been proposed as a complementary index reflecting the balance between ACMSD-mediated diversion to PIC and ongoing QUIN production in microglia [70]. In a CSF study of suicide attempters, Brundin et al. demonstrated that ACMSD variants and activity influence QUIN load and discussed QUIN/PIC (and inversely PIC/QUIN) as potential biomarkers of vulnerability to suicidality and neuroinflammation [70]. Later reviews and quantitative syntheses of tryptophan dysmetabolism in depression and suicide highlight this ratio as an attractive integrative marker of microglial versus astrocytic pathway bias [69].

Taken together, these ratio-based measures consistently outperform single metabolites when it comes to mapping onto clinical phenomena. Across studies, lower KYNA/QUIN and PIC/QUIN and higher 3-HK/KYNA tend to co-occur with greater depressive severity, higher suicidal risk, cognitive impairment, and—in some datasets—poorer treatment response, supporting the idea that KP “balance” rather than any isolated metabolite is the most clinically informative signal.

## 3. Clinical Evidence Across Psychiatric Disorders

### 3.1. From Kynurenine Pathway Dysregulation to Neurocognitive Vulnerability

Beyond serving as markers of immune–metabolic state, emerging evidence suggests that kynurenine pathway (KP) dysregulation may bias core neurocognitive processes that translate biological vulnerability into clinical phenotype. Experimental and translational studies increasingly link shifts in KP balance—particularly QUIN- and 3-HK-dominant profiles relative to KYNA—to altered threat processing, attentional control, and executive regulation across frontostriatal and corticolimbic circuits (Chen et al., 2021; Göy et al., 2025) [74,75]. These effects appear especially salient under stress, where inflammatory or glucocorticoid-driven KP activation may amplify sensitivity to salient or threatening stimuli, impair cognitive filtering, and reduce behavioral flexibility (Vecchiarelli et al., 2016; Jang et al., 2022) [76,77]. Convergent evidence further links KP imbalance to deficits in associative memory, habituation, and cognitive control, suggesting that KP-related neurotoxicity may preferentially disrupt information-processing systems required for adaptive regulation under uncertainty (Chirico et al., 2020; de la Flor and O’Connor, 2025) [78,79]. Importantly, this literature supports the view that KP dysregulation does not map onto discrete diagnoses, but instead modulates transdiagnostic vulnerability dimensions—such as emotional reactivity, attentional bias, and impulsivity—that shape symptom expression across developmental and contextual settings (Comai et al., 2016; Javelle et al., 2021; Christou et al., 2026) [80,81,82].

### 3.2. From Diagnosis to Dimensions: Why a Transdiagnostic Framework

Building on this framework, the following sections examine how KP dysregulation relates to specific symptom dimensions and clinical outcomes across psychiatric disorders. Large-scale meta-analyses have identified kynurenine pathway (KP) abnormalities across major depressive disorder, bipolar disorder, schizophrenia, and suicidality, indicating a robust biological signal despite diagnostic heterogeneity [9,10,42]. Historically, these findings were organized around DSM and ICD categories—linking elevated QUIN to suicidality, reduced KYNA to cognitive deficits in schizophrenia, and altered KYN/TRP ratios to inflammatory mood-disorder subtypes. Yet growing evidence shows that KP disruptions cut across diagnoses and map more closely onto transdiagnostic dimensions such as anhedonia, cognitive impairment, psychomotor slowing, fatigue, and allostatic load. Because categorical labels obscure this distributed biology, the following sections organize KP–clinical associations by symptom constructs rather than diagnoses, aligning with contemporary dimensional models and better reflecting how KP dysregulation manifests across psychiatric illness. To reduce narrative density and support cross-dimensional interpretation, these relationships are synthesized in Table 2.

### 3.3. Suicidality

Among psychiatric phenotypes, suicidality exhibits some of the most robust and consistent kynurenine pathway (KP) alterations. Bryleva and Brundin reviewed evidence of elevated quinolinic acid (QUIN), reduced kynurenic acid (KYNA), and microglial activation in suicidal individuals across cerebrospinal fluid, brain tissue, and peripheral compartments [11]. ACMSD linked to suicidality [70]. Similarly, Serafini et al. summarized findings of altered tryptophan (TRP), kynurenine (KYN), and QUIN in studies of suicidal behavior and treatment-resistant depression [96]. Together, these convergent observations support a model in which dominance of the neurotoxic branch—particularly QUIN elevation—is closely linked to suicidal risk. A 2025 clinical study/meta-analytic synthesis by Demirci et al. found that TRP, KYN and the KYN/TRP ratio are significantly altered in suicide attempters, further supporting KYN/TRP as a systemic risk marker [97]. Preliminary evidence linked plasma KYN to MDD suicide attempters compared to non-attempters [83].

### 3.4. Peripartum Depressive/Mood Dysregulation

Across pregnancy and the postpartum period, profound hormonal and immunological transitions are accompanied by coordinated changes in inflammatory signaling and kynurenine pathway (KP) metabolites. In systematic and empirical studies, Achtyes and colleagues reported that severe postpartum depression is characterized by concurrent immune activation and KP dysregulation, supporting a coupled immune–metabolic phenotype in clinically severe cases [98]. Complementing this work, Quan et al. reviewed evidence linking postpartum depressive symptoms to alterations in KP metabolites, emphasizing how pregnancy- and postpartum-related hormonal and immune shifts shape KP activity and downstream metabolic balance [99].

Longitudinal analyses further suggest that these alterations are phase dependent and may precede clinical symptom emergence. Sha et al. tracked cytokines and KP metabolites across pregnancy and the postpartum period and found that kynurenine and quinolinic acid levels measured during gestation predicted subsequent depressive symptom severity [12]. Consistent with this predictive framework, Modzelewski et al. highlighted second-trimester kynurenine and quinolinic acid levels as promising biomarkers of postpartum depression risk and severity [100]. Together, these findings suggest that dynamic KP shifts during the peripartum period may be associated with increased vulnerability in susceptible individuals and that KP-derived metabolites may have potential as early-warning indicators to support personalized risk stratification and earlier intervention. In parallel, maternal stress and inflammation during pregnancy can be relayed to the developing fetus through placental immune signaling pathways, providing an additional mechanism through which peripartum immune–metabolic perturbations may exert downstream effects [101].

### 3.5. Sleep Dysregulation

Emerging evidence links KP dysregulation to sleep disturbance, suggesting a bidirectional interaction between immune–metabolic signaling and sleep physiology. Experimental work indicates that reducing KYNA under conditions of KP activation may improve sleep architecture, highlighting a potential therapeutic target [87]. Human data similarly show that sleep disturbance is associated with a lower KYNA/QUIN ratio in currently depressed individuals, an effect that remains significant after adjusting for demographic and clinical covariates, whereas no relationship is observed with KYNA/3-HK [88]. In the same cohort, sleep disturbance correlates with elevated C-reactive protein, reinforcing a model in which inflammatory activation shifts KP metabolism toward neurotoxic branches that may disrupt sleep-regulatory circuits. Conceptual syntheses further underscore the relevance of TRP metabolism and KP dynamics in insomnia and its psychiatric and neurological comorbidities [89]. Together, these findings support a mechanistic framework in which inflammation-biased KP flux—particularly reduced KynA relative to QA—contributes to sleep disruption in mood disorders.

### 3.6. Cognitive Dysfunction

Evidence across psychiatric and neurodegenerative disorders indicates that inflammation-driven activation of the kynurenine pathway (KP) contributes to cognitive impairment through a shift toward neurotoxic metabolites. Reviews show that QUIN- and 3-HK-related profiles consistently associate with poorer cognition [102], and emerging work suggests that peripheral KP metabolites may also influence cognition through effects on neurovascular coupling at the choroid plexus [44]. A shared inflammatory etiology for depression and cognitive decline has been proposed, positioning the KP as a potential therapeutic target [103]. Clinically, elevated KYN and higher KYN/TRP ratios correlate with impaired working memory in major depressive disorder [84], while lower KYNA—an endogenous NMDA receptor antagonist—is linked to worse cognitive functioning in bipolar depression [85]. Experimental studies further demonstrate that IDO1-mediated neurotoxic bias contributes to inflammation-induced memory deficits [86], and QUIN has been implicated in cognitive dysfunction in schizophrenia [104]. Collectively, these findings support a model in which increased KYN, 3-HK, and QUIN, together with reduced KYNA, represent a transdiagnostic mechanism of cognitive impairment.

### 3.7. Anhedonia

Anhedonia is a core, disabling symptom of depression across diagnoses, and converging evidence implicates immune–metabolic dysregulation—particularly inflammation-driven alterations in the kynurenine pathway (KP)—in its pathophysiology [105,106,107]. Inflammatory cytokines such as TNF-α increase IDO activity and shift TRP metabolism toward KYN, and individuals with high TNF-α and elevated KYN/TRP ratios show greater depression severity, more pronounced anhedonia, and poorer treatment response [41]. Animal models further demonstrate that inflammation-associated anhedonia is exacerbated by BDNF deficiency [108], consistent with downstream effects of neurotoxic TRYCATs on synaptic plasticity. Clinical data also support a mechanistic role for KP dynamics: in treatment-resistant depression, ECT-induced improvements in anhedonia track with shifts in KP metabolites [109]. Adolescent MDD studies similarly highlight IDO-mediated pathway bias as a contributor to anhedonia, reinforcing the value of dimensional, mechanism-focused approaches [106]. Together, these findings raise the possibility that an immune-related imbalance in neuroprotective (KYNA) versus neurotoxic (QUIN) metabolism promotes dendritic atrophy and anhedonia, positioning KP dysregulation—particularly inflammation-induced IDO activation—as a central biological pathway underlying motivational deficits in depression [53].

### 3.8. Treatment Refractoriness

Neuroprogression describes the chronic, relapsing–remitting trajectory of mood disorders and their associated cognitive and structural deterioration [6,7]. Treatment refractoriness has frequently been associated with elevated inflammatory markers; and several lines of evidence suggest that adjunctive anti-inflammatory treatment may be beneficial in select subsets of treatment-resistant depressive cases [92,93]. In this context, reports of persistent kynurenine pathway (KP) abnormalities across depressive and remitted states raise the possibility of a trait-like immune–metabolic vulnerability that may be relevant to refractory or progressive illness courses [32]. Preclinical meta-analytic work indicates that chronic stress drives a shift toward higher QUIN and lower KYNA, reinforcing a model in which sustained KP imbalance contributes to illness chronicity [110]. These findings have prompted interest in whether KP regulation can inform mechanisms of treatment refractoriness. Clinically, high KYN/TRP and elevated TNF-α cluster with anhedonia, cognitive impairment, and poor SSRI response [107], suggesting a distinct inflammatory–metabolic subtype that may benefit from anti-inflammatory or glutamatergic strategies. In bipolar disorder, poor lithium response has been linked to KP dysregulation [111]. Several KP metabolites also show predictive value for antidepressant response: KYNA has predicted remission with escitalopram [91], baseline AA/ICAM-1 predicted ketamine response in treatment-resistant depression [67], and systematic reviews indicate that ketamine may attenuate inflammatory signaling and reduce activity in the neurotoxic KP arm [90]. Conversely, elevated QUIN has been associated with poorer ketamine outcomes, while higher KYNA may mark individuals more responsive to interventions targeting glutamatergic modulation. Together, these findings position KP biomarkers as promising tools for stratifying treatment response and understanding mechanisms underlying neuroprogression.

### 3.9. Bipolar Disorder: Phase-Dependent KP Dynamics

Bipolar disorder is characterized by marked phase-dependent variation in inflammatory signaling, with meta-analytic evidence indicating higher inflammatory burden during manic and depressive episodes relative to euthymia [112,113]. Emerging data suggest that kynurenine pathway (KP) alterations track these phase-specific immune shifts rather than bipolar diagnosis per se [29,94,112]. During acute mood episodes, KP flux appears biased toward KMO-mediated metabolism [95], resulting in relatively increased production of neurotoxic metabolites such as quinolinic acid and reduced availability of neuroprotective branches. Although findings across studies are heterogeneous, this pattern is consistent with a transient reduction in neurotoxic buffering capacity during periods of heightened immune activation [114]. Repeated or prolonged exposure to such QUIN-dominant states across illness course provides a plausible mechanistic link between inflammatory activity, KP dysregulation, and the progressive cognitive and functional decline observed in a subset of individuals with bipolar disorder, aligning KP dynamics with contemporary models of neuroprogression.

## 4. Confounders and Modifiers of KP Measurements

Interpretation of kynurenine pathway (KP) biomarkers requires careful attention to biological and environmental modifiers that systematically bias pathway flux. Advancing age is associated with higher KYN/TRP ratios and quinolinic acid levels, with frailty amplifying pro-inflammatory KP activation [115,116]. Sex hormone milieu also shapes KP metabolism: hormonal fluctuations across the menstrual cycle, pregnancy, and the postpartum period are accompanied by predictable shifts in KP metabolites [117,118]. Metabolic status represents another major modifier, as obesity is associated with chronic low-grade inflammation that biases tryptophan metabolism toward increased upstream kynurenine production and relatively reduced kynurenic acid formation [119]. Behavioral factors further modulate KP balance; physical activity robustly increases skeletal muscle kynurenine aminotransferase expression, diverting kynurenine toward kynurenic acid and reducing neurotoxic load on the brain, an effect confirmed across acute and chronic exercise paradigms [120,121,122,123].

Emerging evidence also suggests that gut microbiota composition, including probiotic interventions, may influence KP metabolism [124]. More broadly, the KP functions within a larger tryptophan metabolic network that includes parallel pathways. These include gut microbiota-derived indole metabolites, which influence immune signaling and intestinal barrier function, as well as peripherally synthesized serotonin, which does not cross the blood–brain barrier but may affect central processes indirectly via autonomic and vagal afferent pathways [125,126].

In addition to these biological and behavioral modifiers, pharmacologic and developmental factors further shape KP regulation. Several commonly used psychotropic medications influence upstream inflammatory tone, glucocorticoid signaling, and glutamatergic transmission, and may therefore indirectly shift KP balance rather than acting on the pathway as a primary pharmacological target [8]. Developmental stage may also influence KP regulation and its downstream neurocognitive consequences, as age-related differences in immune reactivity, brain maturation, and stress sensitivity likely affect how KP dysregulation is expressed across the lifespan (Dantzer et al., 2008) [127].

Together, these considerations underscore the need to interpret peripheral KP biomarkers in context, with explicit attention to age, hormonal state, metabolic health, behavior, and adjacent tryptophan pathways. Incorporating these modifiers into covariate strategies, sampling protocols, and longitudinal designs is essential to avoid misattributing state- or context-dependent metabolic variation to disease processes alone.

## 5. Conclusions

The kynurenine pathway (KP) offers a mechanistic framework for understanding how immune, metabolic, and stress-related signals are translated into enduring neurobiological vulnerability in psychiatric illness. Rather than functioning as a static biomarker or a simple diversion of tryptophan from 5-HT synthesis, the KP operates as a dynamic, compartmentalized regulator of neurotoxic–neuroprotective balance. Shifts in pathway flux—particularly toward quinolinic acid-dominant states—reflect reduced buffering capacity against inflammatory and excitotoxic stress.

Across mood, psychotic, and stress-related disorders, KP alterations align more closely with illness phase, symptom dimensions, and treatment resistance than with diagnostic boundaries. This pattern supports a transdiagnostic view in which KP profiles index state-dependent vulnerability and cumulative biological burden rather than disease presence alone. Repeated or prolonged periods of QUIN-biased signaling provide a plausible pathway through which fluctuating immune activation may contribute to neuroprogression in susceptible individuals.

Framed in this way, the primary value of KP biomarkers lies not in diagnosis, but in characterizing dynamic risk, resilience, and illness trajectory over time. As methodological standards improve and longitudinal designs mature, KP-informed measures may help bridge molecular processes with clinical staging and treatment stratification. More broadly, conceptualizing the KP as a regulator of buffering capacity reframes inflammation-related psychopathology as a problem of dynamic system imbalance rather than static deficit, offering a biologically grounded lens for future precision approaches in psychiatry.

## Figures and Tables

**Table 1:** Compartmentalization of KP signals and measurement implications.

Compartment	Primary KP Readouts	Key Findings Aligned to ‘Buffering Capacity’	Measurement Caveats	State vs. Trait Signal	Clinical Use-Case	Citations, Meta-Analyses in Bold
Peripheral blood (serum/plasma)	TRP, KYN, KYN/TRP (proxy IDO/TDO activity); 3-HK, KYNA, QUIN; CRP/cytokines as drivers	Inflammation increases TRP → KYN flux; higher KYN/TRP indexes immune-driven shunting. Across mood/psychosis, peripheral patterns suggest a shift toward neurotoxic TRYCAT bias in a subset.	Serum vs. plasma differences; albumin binding alters free TRP; KYN/TRP is an imperfect IDO proxy; diurnal/fasting and renal/hepatic effects.	Mixed: can fluctuate with inflammation (state) but can show persistence across phases in some cohorts (trait-like vulnerability).	Cheap stratification for inflammatory-metabolic subtype; longitudinal tracking alongside symptoms/treatment response.	**Marx et al., 2021 [9]**; **Ogyu et al. [10], 2018**; **Arnone et al., 2018 [42]**; Badawy & Guillemin, 2019 [27]; Felger et al., 2020 [30]; Solmi et al., 2021 [29]
Blood–brain barrier transport interface	LAT1-mediated transport (KYN, TRP); brain availability of KYN as upstream substrate	Peripheral KYN can enter brain and set the ‘supply side’ for downstream neuroactive metabolites—linking peripheral immune activation to central excitatory/inhibitory balance.	Transport competition (other large neutral AAs); illness, stress, and medications can alter transport; inference is indirect unless paired with CSF/brain measures.	Mostly state-dependent (changes with systemic substrate/transport dynamics), but transport capacity may vary by individual.	Interpret peripheral KYN changes in context of CNS exposure; motivates paired central measures in mechanistic studies.	Fukui et al., 1991 [43]; Hall et al., 2019 [17]; Bravi et al., 2025 [44]
CNS—microglia/macrophage arm (neurotoxic branch)	QUIN, 3-HK; KMO activity; markers of oxidative stress/excitotoxicity	Activated immune signaling biases KP toward QUIN/3-HK, increasing excitotoxic and oxidative load—reducing ‘buffering’ and promoting symptom dimensions tied to glutamatergic dysregulation.	QUIN measurement is compartment-sensitive; postmortem vs. in vivo discordance; requires careful sample handling and analytic methods.	Often state-linked to immune activation; repeated episodes/chronic stress may engrain bias (trait-like risk).	Target identification for anti-inflammatory/glutamatergic strategies; links to suicidality and cognitive impairment mechanisms.	Guillemin, 2012 [45]; Lugo-Huitron et al., 2013 [46]; **Bryleva & Brundin, 2017 [11]**; **Arnone et al., 2018 [42]**
CNS—astrocytic arm (neuroprotective branch)	KYNA (NMDA antagonist); KAT activity; KYNA/QUIN ratio	Higher KYNA can buffer excitatory toxicity; reduced KYNA/QUIN ratio suggests reduced buffering capacity and is observed in depressed and remitted phases in some MDD work.	KYNA differs across tissues; central vs. peripheral discordance can be substantial; need matched sampling.	Ratio can show trait-like persistence (e.g., across depressed and remitted phases) while still varying with state.	Candidate trait marker for staging or relapse risk; complements symptom-focused endpoints.	Savitz et al., 2015 [32]; **Skorobogatov et al., 2021 [34]**
CSF/central biofluids	CSF TRYCATs (KYN, KYNA, QUIN) and ratios; cytokines/immune markers	CSF data support that central KP alterations map onto diagnosis and symptom dimensions, strengthening brain relevance vs. peripheral-only inference.	Invasive; sample sizes smaller; assay standardization varies across studies.	More stable than peripheral for some metabolites, but still sensitive to acute inflammation and treatment.	Mechanistic validation of peripheral biomarkers; aligns compartment signals with clinical phenotypes.	Haroon et al., 2020 [21]; **Inam et al., 2023 [37]**; **Almulla et al., 2022 [47]**
Neuroimaging/brain correlates	Associations between KP markers and brain structure/function (e.g., white matter/myelin; neuroimaging correlates)	Links KP imbalance to circuit-level and myelin/white matter phenotypes—bridging molecular buffering capacity to neuroprogression and cognitive outcomes.	Heterogeneous methods; often correlational; require triangulation with biofluid measures.	Likely reflects both state (inflammation-related) and cumulative burden (trait/progression).	Supports staging models and treatment stratification; generates testable mechanistic hypotheses.	Wang et al., 2023 [39]; Ali et al., 2024 [38]

**Table 2:** Clinical dimensions mapped onto KP buffering capacity (state vs. trait).

Clinical Dimension	Hypothesized KP Shift	Core Biomarkers (Typical Compartment)	State vs. Trait	Evidence Snapshot	Translational Implication	Citations (Meta-Analyses in Bold)
Depressive syndrome (core mood)	Inflammation-driven shunt: TRP → KYN (↑KYN/TRP), with downstream bias toward QUIN/3-HK and reduced KYNA/QUIN buffering in a subset	KYN/TRP; KYNA/QUIN; QUIN; CRP/cytokines (blood ± CSF)	Mixed: inflammatory activation is state; ratio shifts may persist (trait-like vulnerability) in some samples	Meta-analytic signal of KP abnormalities across MDD/BD/SCZ; MDD work suggests lower KYNA/QUIN in both depressed and remitted phases in some cohorts.	Subtype identification for anti-inflammatory, glutamatergic, or metabolic interventions; consider longitudinal monitoring.	**Marx et al., 2021 [9]**; **Ogyu et al. [10], 2018**; Savitz et al., 2015
Suicidality	Reduced buffering capacity via KMO-linked excitotoxicity (↑neurotoxic metabolites; ↓protective balance)	KYN/TRP; KYN; QUIN; KMO-related indices (blood/CSF)	Often state-linked (acute risk), but may index enduring vulnerability in high-risk groups	Reviews synthesize evidence linking KP metabolites to suicidal behavior; individual studies show elevated KYN in suicide attempters with MDD and KMO-related vulnerability.	Risk stratification research; targetable node (KMO/KYNA balance) for mechanistic trials.	**Bryleva & Brundin, 2017 [11]**; Brundin et al., 2016 [70]; Sublette et al., 2011 [83]
Cognition (MDD/BD and across disorders)	Neurotoxic bias and neurovascular disruption; lower buffering may impair attention/working memory and executive function	TRYCATs and ratios; choroid plexus/neurovascular correlates (blood/CSF + imaging)	Both: can worsen with episode/inflammation (state) and track chronicity/neuroprogression (trait)	Recent studies link KP metabolites with cognitive dysfunction in MDD and BD; mechanistic animal work supports IDO-dependent neurotoxic metabolism causing memory deficits.	Design cognition-focused endpoints; pair biomarkers with cognitive domains and imaging where feasible.	Pan et al., 2025 [84]; Hebbrecht et al., 2022 [85]; Heisler & O’Connor, 2015 [86]; Bravi et al., 2025 [44]
Sleep disturbance/insomnia	KYN exposure increases sleep disruption; blocking KYNA synthesis can prevent KYN-induced sleep disturbance (preclinical/experimental)	Brain KYNA synthesis; peripheral KYN/TRP; TRYCAT profiles	Primarily state (sleep and inflammation fluctuate), with possible trait coupling in chronic insomnia/depression	Human and preclinical work links sleep disturbance with altered KP metabolism; recent evidence suggests preventing KYNA synthesis blocks KYN-induced sleep disturbance.	Use sleep phenotyping as a sensitive readout of inflammatory-KP perturbation; consider chronobiology controls.	Rentschler et al., 2024 [87]; Cho et al., 2017 [88]; **Sarawi, 2025 [89]**
Treatment-resistant depression/ketamine response	KP profile may index responsiveness; anti-inflammatory + KP modulation as treatment mechanisms	Anthranilic acid; KYNA; KYN/TRP; inflammatory markers (blood)	Both: baseline KP profile may be trait-like predictor; acute changes can be state/treatment-related	Systematic review summarizes ketamine effects on inflammation/KP; metabolomic work suggests KYNA as overlapping biomarker; AA predicted ketamine remission in TRD cohort.	Biomarker-guided stratification for rapid-acting antidepressants; prioritize baseline predictors + early-change markers.	**Kopra et al., 2021 [90]**; Erabi et al., 2020 [91]; Haroon et al. 2018 [92]; Halaris et al. [93], 2020; Murata et al., 2025 [67]
Bipolar mood-state dynamics	Immune–KP coupling varies by episode; cumulative inflammatory load may reduce buffering across illness course	CRP/cytokines; peripheral KP metabolites; episode-phase comparisons	State (episode differences) + trait/progression (between-episode baseline shifts)	Meta-analyses show inflammatory marker differences across mood states; phase-specific studies show KP differences across depressive/manic/euthymic phases.	Support staging/neuroprogression frameworks; consider episode-specific biomarker sampling in trials.	**Solmi et al., 2021 [29]**; **Fernandes et al., 2016 [94]**; Maget et al., 2020 [95]

## Data Availability

No new data were created or analyzed in this study.
